# Formic Acid Formation by *Clostridium ljungdahlii* at Elevated Pressures of Carbon Dioxide and Hydrogen

**DOI:** 10.3389/fbioe.2018.00006

**Published:** 2018-02-12

**Authors:** Florian Oswald, I. Katharina Stoll, Michaela Zwick, Sophia Herbig, Jörg Sauer, Nikolaos Boukis, Anke Neumann

**Affiliations:** ^1^Technical Biology, Institute of Process Engineering in Life Sciences, Karlsruhe Institute of Technology, Karlsruhe, Germany; ^2^Institute of Catalysis Research and Technology (IKFT), Karlsruhe Institute of Technology, Eggenstein-Leopoldshafen, Germany

**Keywords:** *Clostridium ljungdahlii*, high-pressure fermentation, acetogenic bacteria, acetic acid, formic acid, mass transfer

## Abstract

Low productivities of bioprocesses using gaseous carbon and energy sources are usually caused by the low solubility of those gases (e.g., H_2_ and CO). It has been suggested that increasing the partial pressure of those gases will result in higher dissolved concentrations and should, therefore, be helpful to overcome this obstacle. Investigations of the late 1980s with mixtures of hydrogen and carbon monoxide showed inhibitory effects of carbon monoxide partial pressures above 0.8 bar. Avoiding any effects of carbon monoxide, we investigate growth and product formation of *Clostridium ljungdahlii* at absolute process pressures of 1, 4, and 7 bar in batch stirred tank reactor cultivations with carbon dioxide and hydrogen as sole gaseous carbon and energy source. With increasing process pressure, the product spectrum shifts from mainly acetic acid and ethanol to almost only formic acid at a total system pressure of 7 bar. On the other hand, no significant changes in overall product yield can be observed. By keeping the amount of substance flow rate constant instead of the volumetric gas feed rate when increasing the process pressure, we increased the overall product yield of 7.5 times of what has been previously reported in the literature. After 90 h of cultivation at a total pressure of 7 bar a total of 4 g L^−1^ of products is produced consisting of 82.7 % formic acid, 15.6 % acetic acid, and 1.7 % ethanol.

## Introduction

Nowadays, most bulk chemicals are still based on fossil fuels, such as crude oil and natural gas. It is consensus that, due to dwindling resources and climate change, it is necessary to develop sustainable methods for the production of industrially relevant chemicals. In recent years, industrial exhaust gases, such as steel mill off-gas (Köpke et al., 2011) and synthesis gas (syngas), a mixture of H_2_, CO, and CO_2_, from gasification of biomass and waste streams, such as sewage sludge and municipal waste (Hammerschmidt et al., 2011; Rokni, 2015), as well as other C_1_ molecules came into focus as interesting substrates for biotechnological applications (Daniell et al., 2012; Bengelsdorf et al., 2013). Syngas fermentation uses acetogenic bacteria, a class of bacteria using a unique pathway (Müller, 2003) to combine two molecules of CO or CO_2_ *via* subsequent reactions into one molecule of acetyl-CoA (Diekert and Wohlfarth, 1994). Further conversion of acetyl-CoA yields acetate, ethanol, butyrate, butanol or 2,3-butandiol as natural products of this pathway. Of these, the formation of C_2_ molecules (acetate and ethanol) has the highest energy gain for acetogenic bacteria (Bengelsdorf et al., 2013) which are, therefore, the preferred products with reported concentrations of up to 59.3 g L^−1^ acetate (Kantzow and Weuster-Botz, 2016). A detailed view of the metabolic reactions of acetogenic bacteria can be found in Schuchmann and Müller (2014) or Bengelsdorf et al. (2013). Formic acid is a main intermediate of this pathway which has multiple industrial and commercial applications, such as feed preservation, textile and leather processing, latex coagulation, deicing of airfields, waste gas treatment and as a substitute for other inorganic and organic acids in cleaner formulations. Being a main product of anaerobic syngas fermentation, acetate can be used as carbon source by various organisms to produce substances of higher value. Interlinking anaerobic acetic acid production with aerobic production of malic acid (Oswald et al., 2016) or diesel fuels (Hu et al., 2016) are already shown feasible.

One parameter that limits productivity and growth of acetogenic microorganisms is the aqueous solubility of the sparingly soluble gases carbon monoxide and hydrogen. To achieve high productivity with those substrates, the mass transfer rate from the gaseous to the liquid phase is one of the limiting steps (Worden et al., 1997). Common approaches to enhance gas–liquid mass transfer are increasing the volumetric power input ($PV_{L}^{-1}$) or the volumetric gas feed rate (${\dot{V}}_{\text{g}}V_{L}^{-1}$). Both leads to increased gas–liquid interfacial area due to smaller bubbles and to increased bubble residence times, thus increasing the volumetric gas–liquid mass transfer coefficient (*k*_L_*a*) (Bredwell and Worden, 1998). This approach, however, may not be economically feasible when producing low value products such as fuels (Bredwell et al., 1999). Gas–liquid mass transfer is commonly described by

(1) $$\frac{\text{d}c}{\text{d}t}=k_{\text{L}}a(c*-c)\text{.}$$

with (c*−*c*) being the difference of saturation concentration and actual concentration of a compound at process conditions. Therefore, another possibility to increase gas–liquid mass transfer is to increase the solubility of the gaseous compound itself. This can be achieved by increasing the partial pressure of desired substances (Schmidt and Cooney, 1986). Applying Henry’s law of solubility of gaseous compounds in liquids to equation (1) results in (Vega et al., 1989a)

(2) $$\frac{\text{d}c_{i}}{\text{d}t}\text{=}k_{\text{L}}a_{i}H_{i}\left(p_{i}^{\text{G}}-p_{i}^{\text{L}}\right)\text{.}$$

Where *c_i_* is the liquid concentration of compound *i* in M, *H_i_* is the Henry’s solubility coefficient of *i* in M bar^−1^ and $\text{(}p_{i}^{\text{G}}-p_{i}^{\text{L}}\text{)}$ is the driving force expressed as difference in partial pressure of *i* in bar.

Some work addressing that approach has been done in the 1980s using *Clostridium ljungdahlii* (Vega et al., 1989b), *Blautia producta* (Vega et al., 1989a) and *Clostridium* sp. ATCC 29797, later described as *Terrisporobacter glycolicus* (Schmidt and Cooney, 1986). Vega et al. (1989b) report prolonged lag-phases in bottle experiments with *C. ljungdahlii* under increasing initial *p*_CO_ and $p_{{\text{H}}_{2}}$ up to 2.53 bar absolute pressure and relate this to inhibition caused by increased levels of dissolved carbon monoxide. Comparable findings are reported for bottle experiments with *B. producta* growing on a gas mixture of 80 % CO and 20 % CO_2_ (Vega et al., 1989a). In 1993, the Department of Chemical Engineering of the University of Arkansas filed a report to the United States Department of Energy in which they describe continuous running cultivations with *C. ljungdahlii* in a stirred tank reactor (STR) under increased pressure with total system pressures up to 10.34 bar. They find that when the reactor is pressurized stepwise once the biomass starts to grow, no inhibitory effect of increased carbon monoxide partial pressure can be observed. Immediate pressurization of the STR resulted in reduced growth and productivity (Department of Chemical Engineering, University of Arkansas, 1993). For *B. producta*, Ko et al. (1989) calculated an inhibitory *p*_CO_ of 0.81–1.01 bar employing a modified Monod-Model. Cultivation with only carbon dioxide and hydrogen in the gas stream would circumvent inhibition caused by carbon monoxide. For *Acetobacterium woodii*, studies with gas mixtures devoid of carbon monoxide can be found in Demler (2012) and Kantzow and Weuster-Botz (2016), which investigate the effect of increased $p_{{\text{H}}_{2}}$ but leave the effect of carbon dioxide partial pressure out of their consideration. Therefore, this work focuses on the effects of increased pressure on growth and product formation of *C. ljungdahlii* with a gas composition devoid of carbon monoxide. The aim of this work is to investigate if mass transfer limitation can be overcome and whether or not complete substrate utilization is possible by applying elevated pressure. Experiments in 1.5 L-scale are used to scale-up process parameters to 2.5 L-scale, where experiments at absolute system pressures of 1, 4, and 7 bar are conducted.

## Materials and Methods

### Culture Medium

If not stated differently, all chemicals are purchased from Carl-Roth (Germany). The organism used for this work is *C. ljungdahlii* DSM13528 which was kindly provided by the group of Peter Dürre, University of Ulm. Medium used for cultivation of *C. ljungdahlii* for both flask and bioreactor cultivation is based on Tanner (2007). It is prepared using strict anaerobic techniques and the detailed composition with 0.33 g ammonium chloride per liter medium can be found elsewhere (Oswald et al., 2016). The pH is adjusted to 5.9 using KOH before bottling. Bottles are anaerobized using a gas mixture containing 20 vol-% carbon dioxide in nitrogen (Air Liquide, France). After autoclaving at 121 °C, 1 g Cystein-HCl monohydrate and 5 g fructose per liter are added.

### Cultivations in 1.5 L-Scale

Fermentations are conducted at 37 °C in Minifors bench-top stirred tank reactors (Infors-HT, Switzerland) as described in Oswald et al. (2016) but without a foam probe. Contraspum A 4050 HAC (Zschimmer und Schwarz, Germany*)* is used as an anti-foaming agent and one drop is added to the cultivation if found necessary. The gas flow rate in this scale is 43 mL min^−1^ (0.029 vvm). Gas composition used in this work is 53.3 vol-% H_2_ and 26.7 vol-% CO_2_ in nitrogen (Air Liquide, France). The headspace of the bioreactor is at atmospheric pressure. A stirrer set-up of two Rushton-Turbines and baffles inside the vessel ensures gas-liquid mixing at 800 min^−1^. Medium for bioreactor cultivations is prepared as described in Oswald et al. (2016). Bioreactors are inoculated with 10 % of their final volume of a pre-culture grown for 48 h at 37 °C with 5 g L^−1^ fructose as carbon and energy source.

### Scale-Up to 2.5 L and Elevated Pressure

**Figure 1:**
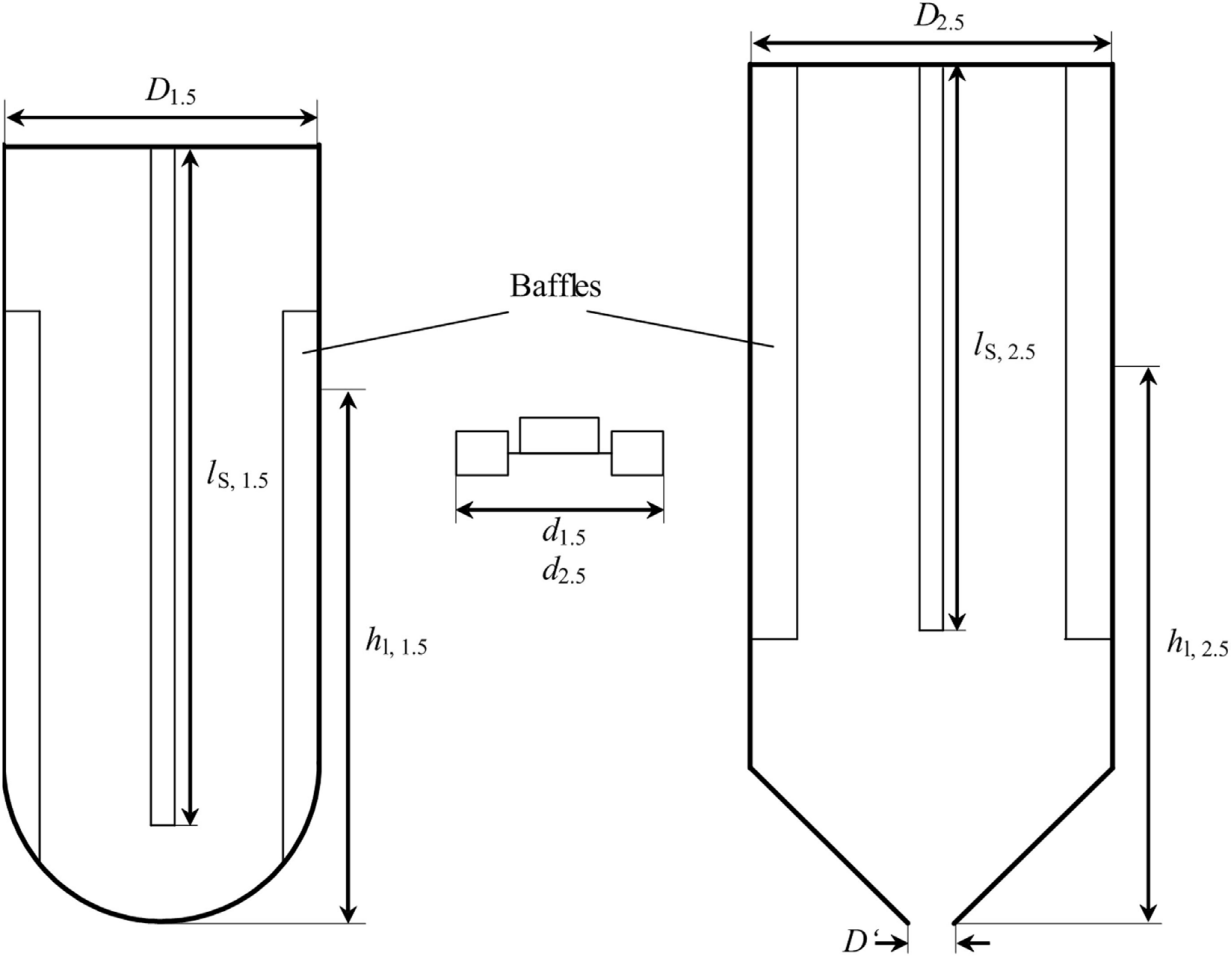
Schematic drawing of the stirred tank reactor used for 1.5 L scale (left) and 2.5 L scale (right). Between both is one of the two Rushton-Turbines used in each of the reactors. *D*_1.5_ = 110 mm, *d*_1.5_ = 46 mm, *l*_S, 1.5_ = 235 mm, *h*_l, 1.5_ = 176 mm, *D*_2.5_ = 126 mm, *d*_2.5_ = 52.7 mm, *l*_S, 2.5_ = 240 mm, *h*_l, 2.5_ = 234.6 mm. Filling level *h*_l_ is without installed equipment (stirrer shaft, baffles, probes, sparger).

To ensure comparability of the results from 1.5 L-scale and 2.5 L-scale, geometric similarity between both scales needs to be given and important dimensionless numbers describing the process are kept constant. Figure 1 shows a schematic drawing of both reactor scales. The bioreactor for experiments in 2.5 L-scale is a stainless steel, double jacket vessel (VEB CLG – Chemieanlagenkombinat Leibzig-Grima, Germany) with an inner diameter *D*_2.5_ of 126 mm and a total volume of 4 L. It has been formerly used in high-pressure chemical catalysis. The total height of the internal space is 349 mm with a conical bottom of 54 mm height. For 1.5 L-scale experiments, a glass vessel from Infors with an inner diameter *D*_1.5_ of 110 mm, a total height of 270 mm, and a hemispherical bottom is used. With the fixed dimensions of the stainless steel vessel, the only possibility to keep *d*/*D* constant is by adjusting the stirrer diameter *d*_2.5_. Using the stirrer diameter from 1.5 L-scale *d*_1.5_ = 46 mm and the inner diameter of the glass vessel, *d*_1.5_/*D*_1.5_ calculates to 0.418. Transferring this to the measures of 2.5 L-scale results in *d*_2.5_ of 57.5 mm. The stirrer of the 2.5 L-scale is a proportional magnification of the stirrer in 1.5 L-scale.

The geometric similarity representing stirrer positions would be 51.5 and 157 mm above the deepest part of the stainless steel vessel. As Figure 1 shows, the stirrer shaft (BüchiGlasUster, Switzerland) of the bigger scale does not reach into the conical part of the vessel. So a position of 51.5 mm above the deepest part of the reactor is not possible. Hence, the compromise is to calculate the stirrer positions, filling volume, and filing level from the cylindrical part of the vessel neglecting the conical bottom. Resulting stirrer positions are 105.5 mm and 211 mm above the deepest part of the vessel (including the conical part) and total filling level *h*_l, 2.5_ is 234.6 mm. This also affects the filling volume of the reactor without installed equipment, which is the volume of the 1.5 L-scale multiplied with (*D*_2.5_/*D*_1.5_)^3^ plus the volume of the conical part yielding a volume of 2.51 L.

Ju and Chase (1992) summarize different scale-up strategies from literature. Of those, the strategies used here are geometric similarity as well as constant *k*_L_*a*-value, stirrer speed and Ne number. Schlüter et al. (1992) state that if volumetric power input $PV_{L}^{-1}$ and volumetric gas feed rate ${\dot{V}}_{\text{g}}V_{L}^{-1}$ are of the same value in both scales, then the volumetric mass transfer coefficient has the same value as well. Therefore, power input for 1.5 L-scale is measured as the difference in power uptake of stirring in air and stirring in 1.5 L of water and a gas feed rate of 0.029 vvm. Keeping Ne constant allows calculating the necessary stirrer speed at $PV_{L}^{-1}$ = constant to 757 min^−1^. The gas feed rate of the larger scale at one bar of absolute pressure calculates to 72 mL min^−1^.

**Figure 2:**
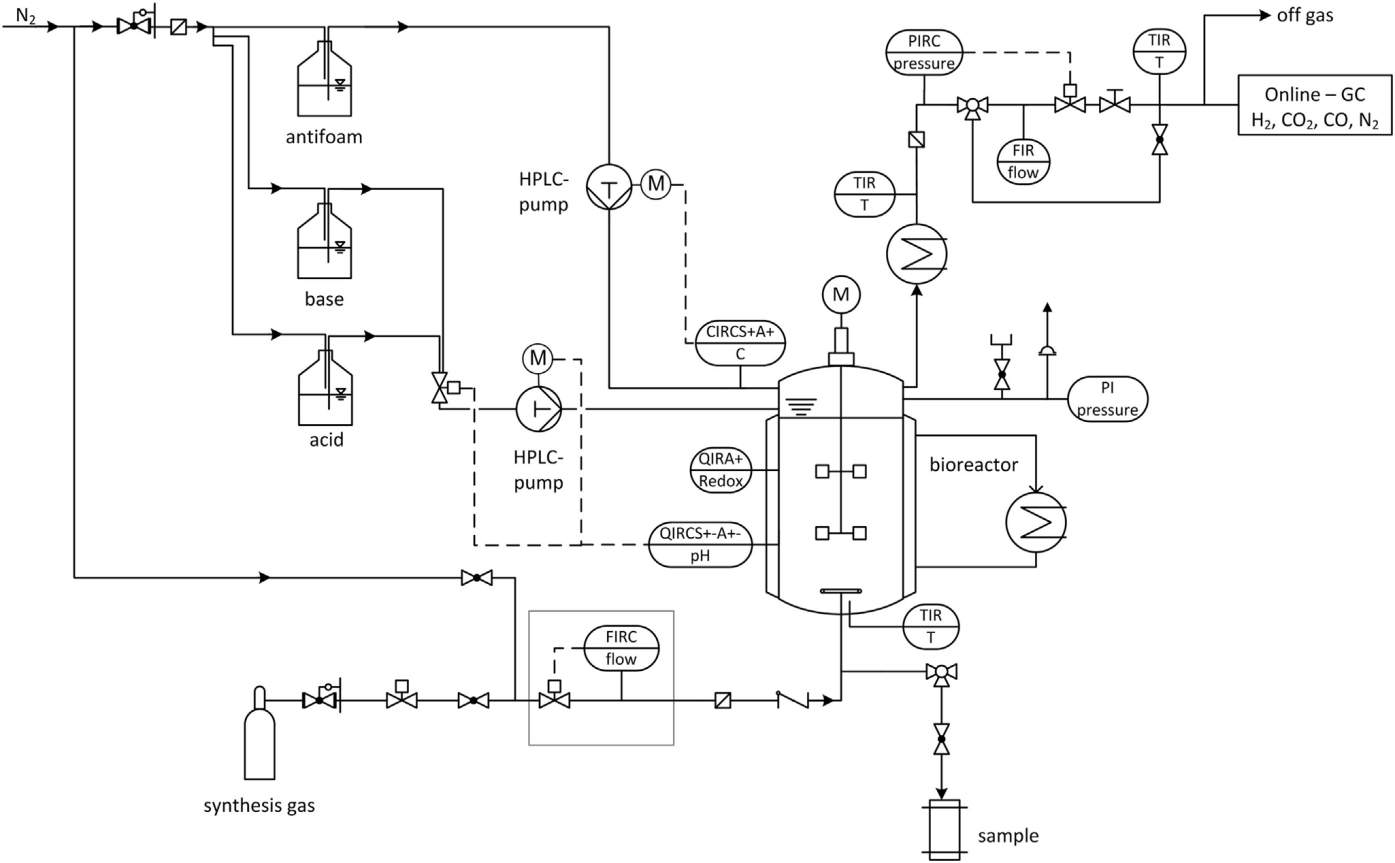
Flow chart of stirred tank reactor (STR) used for elevated pressure cultivations with installed periphery. FIRC, flow indication, recording and control; QIRCS+−A+−, pH indication, recording and control (pH probe); QIRA+, redox potential indication and recording (redox probe); CIRC, current indication, recording and control (AF-electrode); TIR, temperature indication and recording; PIRC, pressure indication, recording and control; FIR, flow indication and recording; PI, pressure indicator.

The following pressure stages are investigated (in absolute pressure): 1, 4, and 7 bar at which the volumetric amount of substance flow rate $\dot{n}V_{L}^{-1}$ is kept constant for all experiments. Gas is dispersed inside the reactor by a sintered metal plate at the end of a 1/4”-tube and pressurization of the bioreactor starts immediately after inoculation *via* closing the installed pressure regulator until the desired pressure is achieved. The medium composition is the same as for 1.5 L-scale and cultivation volume is 2.5 L. Each experiment is seeded with 10 % of the final volume of a 48 h, fructose grown culture. Cultivation temperature and pH-value are set to 37 °C and 5.9, respectively, and the stirrer speed and gas feed rate from above are applied. Maximum cultivation time is 90 h. Figure 2 shows the flow chart of the high-pressure reactor and its installed periphery. Mass flow of feed gas is controlled and regulated by a Coriolis force mass flow controller (Bronkhorst, Netherlands) and mass flow meter (MFM, Bronkhorst, Netherlands). Pressures higher than 1 bar absolute are regulated by a pressure regulator and sensory valve (Bronkhorst, Netherlands) positioned in the off-gas line behind the MFM. Off-gas composition is measured by a gas chromatograph (Shimadzu, Japan). Cultivation temperature is maintained *via* the double jacket and a thermostat (Haake, Germany) and the off-gas is cooled to minimize water loss through evaporation. A HPLC-pump (Bischoff, Germany) is necessary to control the addition of pH adjustment solutions through capillary tubes at pressures above 1 bar absolute. A six-port valve allows switching between 4 M H_3_PO_4_ and 4 M KOH. Both are kept under a nitrogen atmosphere. A second HPLC-pump (Bischoff, Germany) adds anti-foam agent (Zschimmer und Schwarz, Germany) in case the AF-electrode gives a signal. Gas streams are sterile filtered by a 0.2 μm sinter metal filter (Swagelok, Germany) before the feed gas enters the reactor and before the off-gas enters the pressure sensor. A check valve between the reactor and feed gas filter prevents liquid from the reactor to block the filter. ORP-probe (Corr Instruments, USA) and pH-probe (Corr Instruments, USA) for pressurized applications are mounted horizontally at half height through the sides of the reactor. The pH-probe is disinfected with isopropanol. It is installed after steam sterilizing the reactor at 121°C and before it is filled with medium due to a maximum temperature tolerance of the pH-probe of 80 °C.

Analytics for all experiments with regard to off-gas and offline samples is conducted as described in Oswald et al. (2016). Formic acid concentration is determined using an enzymatic assay from Roche yellow line.

## Results

Cultivations in 1.5 L-scale are conducted to establish a baseline for performance at atmospheric pressure with carbon dioxide and hydrogen as carbon end energy sources. The conditions of 1.5 L-scale are then transferred into 2.5 L-scale where experiments at absolute system pressures of 1, 4, and 7 bar are conducted.

**Figure 3:**
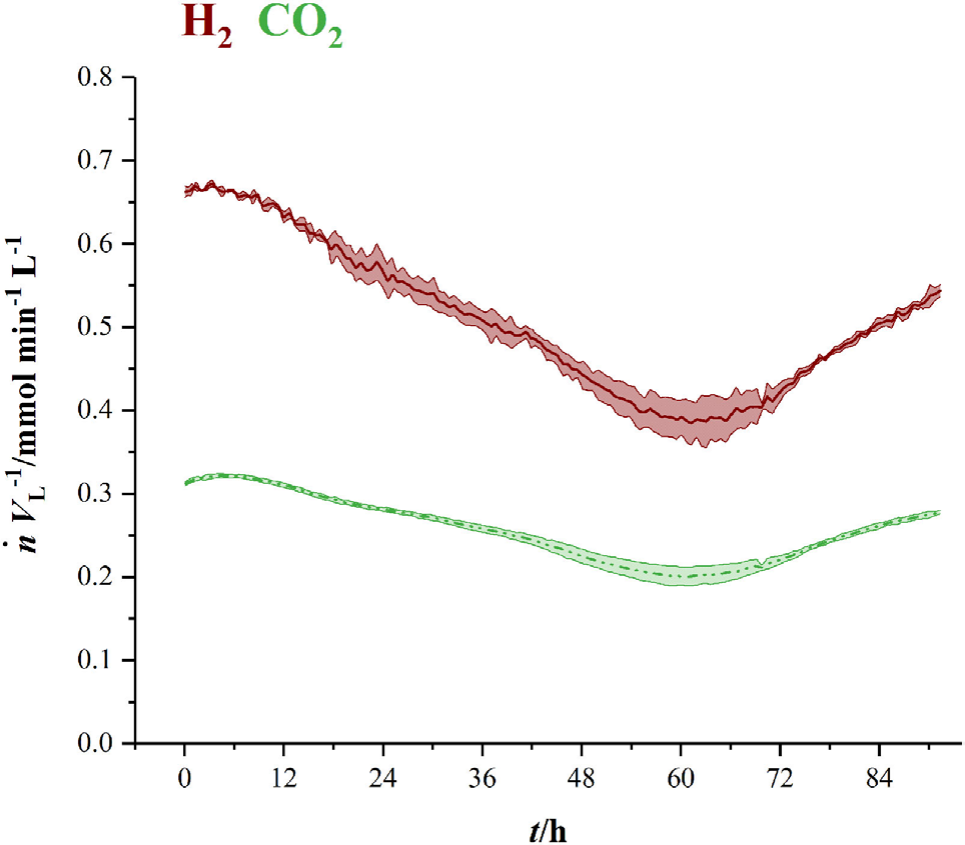
Amount of substance flow rates per liter medium for hydrogen (red, solid) and carbon dioxide (green, dashed) in the off-gas of 1.5 L-scale. Results are average values of three experiments. Standard deviation is indicated by the light colored area around the average lines.

**Figure 4:**
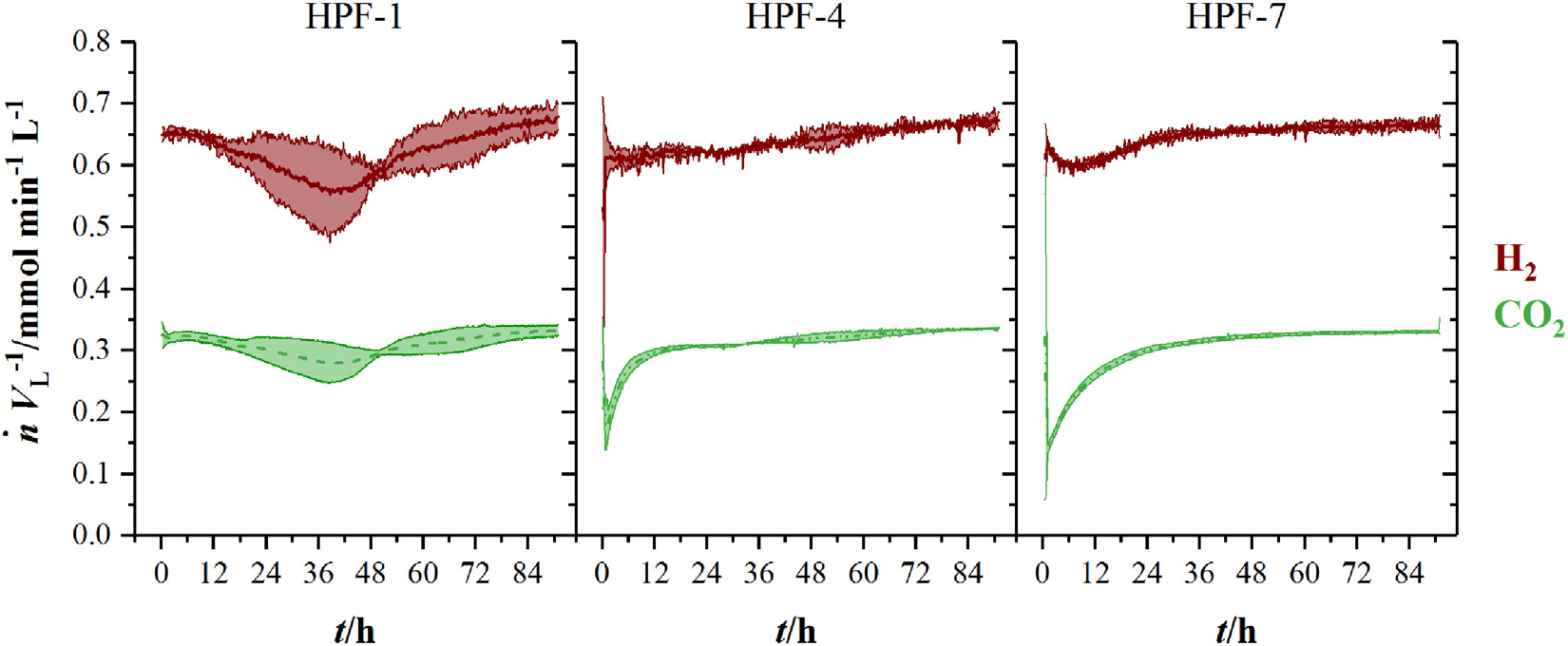
Amount of substance flow rates per liter medium for hydrogen (red, solid) and carbon dioxide (green, dashed) in the off-gas of 2.5 L-scale experiments. Results are average values of three experiments for HPF-1 and HPF-7 and two experiments for HPF-4. Numbers behind HPF indicate the absolute pressure of the fermentation in 2.5 L-scale. Standard deviation is indicated by the light colored area around the average lines.

Figure 3 shows the volumetric amount of substance flow rates $\dot{n}V_{L}^{-1}$ in the off-gas of three fermentations in 1.5 L-scale. Initial carbon dioxide and hydrogen flow rates in the off-gas of 0.3 mmol min^−1^ L^−1^ (CO_2_) and 0.63 mmol min^−1^ L^−1^ (H_2_) continuously decrease until 60 h (CO_2_) and 63 h (H_2_), where they reach their local minimum of 0.18 mmol min^−1^ L^−1^ (CO_2_) and 0.35 mmol min^−1^ L^−1^ (H_2_), respectively. Here, *C. ljungdahlii* consumes 45 % of the ingoing hydrogen and 38 % of the ingoing carbon dioxide. From that point on, uptake rate of both gases decreases and reaches off-gas flow rates of 0.25 mmol min^−1^ L^−1^ for carbon dioxide and 0.49 mmol min^−1^ L^−1^ for hydrogen at the end of fermentation. Transferring the 1.5 L-scale to 2.5 L for pressurized experiments while keeping $\dot{n}V_{L}^{-1}$ constant resulted in decreasing volumetric flow rates with increasing pressure. The volumetric amount of substance flow rates in off-gases from 2.5 L-scale are shown in Figure 4. Off-gas data from high-pressure fermentation at 1 bar of absolute pressure (HPF-1) shows a development comparable to the data of 1.5 L-scale in Figure 3. The three experiments summarized in HPF-1 (Figure 4) show some degree of variation in the development of hydrogen and carbon dioxide in the off-gas and thus have a higher standard deviation than the data from 1.5 L-scale. Off-gas data from high-pressure fermentations with 4 bar of absolute pressure (HPF-4) show a similar development as at 7 bar of absolute pressure (HPF-7). Hydrogen and carbon dioxide have a sharp decrease once the set pressure is reached and asymptotically increase to the initial flow rate. Pressure build up took 30 min for HPF-4 and 75 min for HPF-7. Complete consumption of substrates could not be achieved in any of the conducted fermentations.

**Figure 5:**
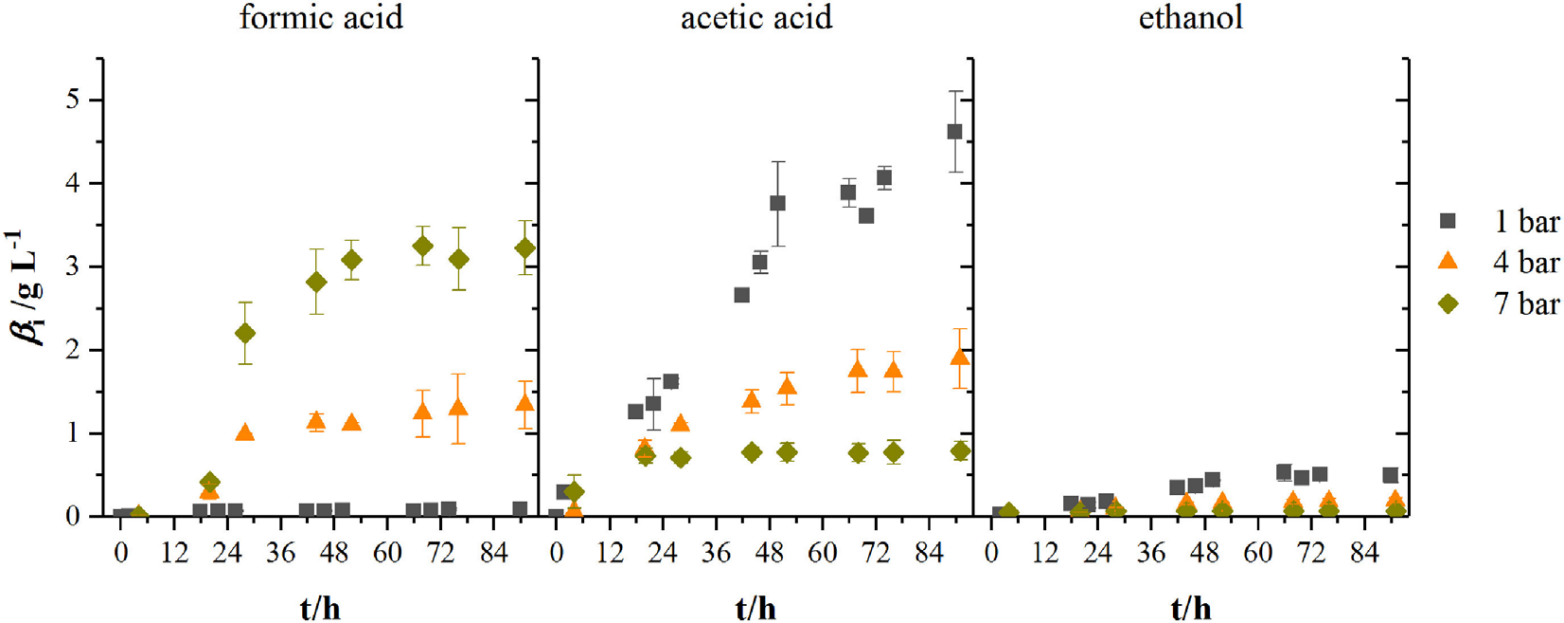
Development of product concentrations for formic acid, acetic acid, and ethanol at different headspace pressures. Results are average values of three experiments for HPF-1 (dark gray squares) and HPF-7 (dark yellow diamonds) and two experiments for HPF-4 (orange triangles). Numbers behind HPF indicate the absolute pressure of the fermentation in 2.5 L-scale.

Figure 5 shows the development of product concentrations over the course of the fermentations in 2.5 L-scale while Table 1 lists resulting product concentrations and consumed amount of substrates per liter reactor volume of experiments in 1.5 L-scale together with the results from HPF-1, HPF-4, and HPF-7. At atmospheric pressure, ethanol and acetic acid are the main products, their concentrations decrease with increasing pressure whereas formic acid concentration increases from final concentrations of 0.09 to 1.34 g L^−1^ at 4 bar and 3.23 g L^−1^ at 7 bar absolute pressure. Acetic acid production starts in all experiments immediately after inoculation while formic acid formation has its strongest increase between 12 and 28 h. The consumed amounts of substrates per liter reactor volume at atmospheric pressure in 2.5 L-scale are a third of the amounts in 1.5 L-scale. Whereas the overall consumption ratio *E* (consumed amount of substance divided by the total fed amount of substance in per cent) is about half the value from 1.5 L-scale. Comparing the biomass-specific uptake rates for hydrogen (*q*_H2_) and carbon dioxide (*q*_CO2_) shows only differences in the uptake of hydrogen. Experiments in HPF-1 show about twice the maximum uptake rates for hydrogen than the ones found for 1.5 L-scale. However, the replicates in HPF-1 divert significantly from each other as can be seen in the off-gas data in Figure 4. This results in rather high standard deviation. Despite the differences in overall consumption, the product yields based on consumed substrates (H_2_ and CO_2_) are quite similar with 0.67 g g^−1^ in 1.5 L-scale and 0.64 g g^−1^ in HPF-1. For experiments at 4 and 7 bar, no consumption data are available. As can be seen from the off-gas data in Figure 4, under pressurized conditions, no reasonable values for consumed substrates can be determined since the data resembles saturation curves for carbon dioxide at elevated pressures. Therefore, yields are additionally calculated based on totally fed substrates (aka overall *Y*_P/S_). In 1.5 L-scale, an overall *Y*_P/S_ of 0.15 g g^−1^ is achieved whereas in 2.5 L-scale for HPF-1, HPF-4, and HPF-7 overall *Y*_P/S_ values of 0.05 g g^−1^, 0.04 g g^−1^, and 0.04 g g^−1^ are achieved, respectively. No significant increase in OD is observed at elevated pressures (data not shown).

**Table 1:** Average values for products and consumed substrates from cultivations of *Clostridium ljungdahlii* with hydrogen and carbon dioxide as sole energy and carbon source at different pressures after 90 h of cultivation.

Set-up	*β*_formic acid_ (g L^−1^)	*β*_acetic acid_ (g L^−1^)	*β*_EtOH_ (g L^−1^)	$c_{H_{2},R}$ (mol L^−1^)	$c_{{\text{CO}}_{2},R}$ (mol L^−1^)	$E_{H_{2}}$ (%)	$E_{{\text{CO}}_{2}}$ (%)	$q_{H_{2},max}$ (mmol min^−1^ g^−1^)	$q_{{\text{CO}}_{2},max}$ (mmol min^−1^ g^−1^)
1.5 L	0.03 ± 0.00	9.30 ± 2.30	2.81 ± 0.13	1.00 ± 0.06	0.40 ± 0.02	25.63 ± 1.32	21.52 ± 0.90	2.40 ± 0.10	1.00 ± 0.05
HPF-1	0.09 ± 0.09	4.29 ± 0.67	0.42 ± 0.15	0.32 ± 0.08	0.14 ± 0.03	12.17 ± 3.07	11.17 ± 2.79	4.56 ± 4.69	1.05 ± 0.61
HPF-4	1.34 ± 0.28	1.90 ± 0.36	0.20 ± 0.03	N/A	NA	N/A	N/A	N/A	N/A
HPF-7	3.23 ± 0.32	0.79 ± 0.11	0.07 ± 0.01	N/A	N/A	N/A	N/A	N/A	N/A

*Numbers behind HPF indicate the absolute pressure of the fermentation in 2.5 L-scale. In 1.5 L-scale, the absolute pressure is 1 bar. $c_{H_{2},R}$, consumed amount of hydrogen per liter reactor volume; $c_{{\text{CO}}_{2},R}$, consumed amount of carbon dioxide per liter reactor volume; $E_{H_{2}}$, consumption ratio of hydrogen as consumed amount of hydrogen in per cent of total amount of ingoing hydrogen; $E_{{\text{CO}}_{2}}$, consumption ratio of carbon dioxide as consumed amount of carbon dioxide in percent of total amount of ingoing carbon dioxide; $q_{H_{2},\text{max}}$, maximum biomass-specific uptake rate of hydrogen; $q_{{\text{CO}}_{2},\text{max}}$, maximum biomass-specific uptake rate of carbon dioxide; N/A, data not available. Average values of three bioreactors per experimental set-up except for HPF-4. For this, values are averages of two bioreactors*.

Due to the fact that in 2.5 L-scale the pH-probe is installed after the reactor is sterilized, contamination with *Bacillus cereus* can be found in all HPF cultivations. Blank cultivations without *C. ljungdahlii* inoculum but with the 0.1 g L^−1^ of fructose carried over from the pre-culture yield the same degree of contamination as the samples from experiments with *C. ljungdahlii* cells. Neither growth nor products can be found in these blank cultivations.

## Discussion

Experiments in 1.5 L-scale are conducted at a *k*_L_*a* value of 10.2 10^−3^ s^−1^ (measured for oxygen in medium, data not shown) and since $PV_{L}^{-1}$ and ${\dot{V}}_{\text{g}}V_{L}^{-1}$ are kept constant, the mass transfer coefficient should have the same value in 2.5 L-scale (Schlüter et al., 1992). Nevertheless, both scales do not show complete geometric similarity as outlined in the Section “Materials and Methods.” Those discrepancies from geometric similarity may explain the observed deviations in product concentration and substrate consumption between 1.5 and 2.5 L-scale at 1 bar absolute pressure. Supporting this are the *Y*_P/S_ values based on consumed substrates. For both scales, this yield is quite similar with the one from HPF-1 being only 4 % lower than the one found in 1.5 L-scale. That means that in both cases metabolic activity is similar since the same ratio of consumed substrates end up in products. Of far more interest in assessing the whole experimental set-up for 2.5 L-scale is the yield based on totally fed substrates during the fermentation. This value shows the overall conversion efficiency of the set-up and one aim of improving every process should be to bring this value as close to the yield based on consumed substrates as possible. For the case at hand, 15 % of gaseous substrates fed in 1.5 L-scale end up in products while in 2.5 L-scale only 5 % and at elevated pressures 4 % can be found in products. We interpret the high conformity of the values for 2.5 L-scale as indication that the found differences in substrate consumption and product concentration between scales at atmospheric conditions are due to incomplete geometric similarity and independent of absolute process pressure.

**Figure 6:**
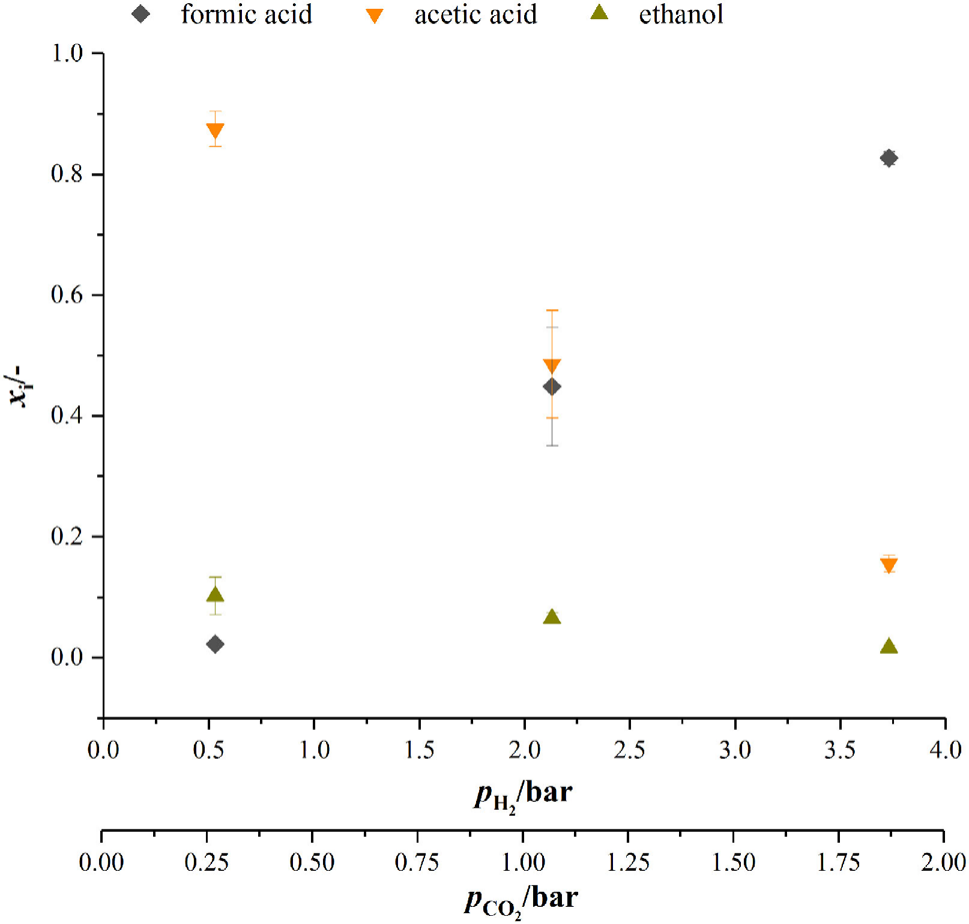
Amount of substance ratio for products at the end of cultivations at elevated pressure. dark gray diamonds, formic acid; orange upturned triangles, acetic acid; dark yellow triangles, ethanol.

When looking at the product spectrum of the conducted experiments in Figure 5, the main thing that jumps the eye is that with increasing pressure the spectrum is shifted toward formic acid formation. At a total pressure of 7 bar almost no ethanol and only 0.8 g L^−1^ acetic acid are produced over the course of fermentation (see Figure 5) while a total of 3.2 g L^−1^ of formic acid is produced. Figure 6 shows the amount of substance ratios (*x*_i_ = *c_i_*/Σ*c_i_*) of the products at the end of our cultivations at elevated pressure. It seems that at a $P_{H_{2}}$ of 2.13 bar (4 bar total pressure) formic acid and acetic acid are produced in equimolar amounts while at a $P_{H_{2}}$ of 3.73 bar (7 bar total pressure) values of *x* for formic acid and acetic acid seem to be inverted compared to experiments at atmospheric conditions. The data also suggest that in the range of $p_{{\text{H}}_{2}}$ from 0.5 to 3.37 bar (corresponding $p_{{\text{CO}}_{2}}$ from 0.25 to 1.9 bar) there might be a linear relationship between *x*_i_ and the substrate partial pressure. Increased formic acid production at elevated pressures with H_2_/CO_2_ is described by Bleichert and Winter (1994) for pure cultures of *Methanobacterium formicicum* and *Methanobacterium palustre* as well as for mixed cultures from sewage sludge at hydrogen partial pressures of more than 2 bar. Kantzow and Weuster-Botz (2016) and before them Peters et al. (1999) show that formic acid formation is linked to the hydrogen partial pressure in *A. woodii*. By shifting the hydrogen partial pressure from 1.4 to 2.1 bar, they increased final formic acid concentration after 74.4 h of cultivation from 4.2 to 7.3 g L^−1^ and increased the yield of formic acid per gram substrates fed of about 67 % (Kantzow and Weuster-Botz, 2016). Peters et al. (1999) report an increase in formic acid production for bottle experiments of 0.5 mM per 0.1 bar increase in initial $p_{{\text{H}}_{2}}$. In *A. woodii*, the hydrogen dependent carbon dioxide reductase (HDCR) catalyzes the hydrogenation of CO_2_ with molecular hydrogen (Schuchmann and Müller, 2013) while in *C. autoethanogenum*, a close relative to *C. ljungdahlii*, the direct hydrogenation of CO_2_ with H_2_ is one of three possible reactions of the hydrogenase-formate dehydrogenase complex funneling CO_2_ into the methyl branch of the Wood–Ljungdahl pathway (Wang et al., 2013). The next reaction links formic acid and tetrahydrofolic acid (THF) in an ATP-consuming reaction. This poses as a bottleneck in the methyl branch since the specific activities of this and the following reactions of the methyl branch are lower than the one of the hydrogenase-formate dehydrogenase complex (Wang et al., 2013). As for the effect of increased substrate partial pressure, dissolved CO_2_ is in balance with the concentration of ${\text{HCO}}_{3}^{-}$. Carbon dioxide can freely diffuse through the cellular membrane (Gutknecht et al., 1977) and immediately dissociates into ${\text{HCO}}_{3}^{-}$ and H^+^, thus acidifying the cytoplasmic pH. One consequence of that is a reduction in membrane potential (Eigenstetter and Takors, 2017) which results in reduced ATP yield from ATPase activity. Since acetogenic organisms are already at the energetic limit of life (Schuchmann and Müller, 2014) at these conditions, ATP formation is not high enough to provide enough energy for formyl-THF formation (Yang and Drake, 1990; Kantzow and Weuster-Botz, 2016). This yields to an accumulation of formic acid at increased substrate partial pressures and is in accordance with our finding that biomass formation is severely inhibited at increased partial pressures of carbon dioxide. Supporting this model further is the fact that in the work of Kantzow and Weuster-Botz (2016) with *A. woodii* no such severe effects are reported. The reason for this is that energy conservation is driven by a transmebrane gradient of sodium ions in *A. woodii* instead of a proton gradient (Spruth et al., 1995; Biegel and Müller, 2010). Therefore, a drop in internal pH will not result in immediate reduction of ATP formation.

Unfortunately, the only publications that state results from experiments with *C. ljungdahlii* at elevated substrate pressures so far do not report if formic acid production is increased at higher pressures. This may be because no formic acid is produced when working with CO containing gases at elevated pressures or more likely because the authors did not check for formic acid in their sample analytics. However, since we could not observe significant growth at 4 and 7 bar even without CO in the gas atmosphere growth inhibition at elevated pressures seems not to be linked to inhibitory effects of carbon monoxide alone, as reported by Vega et al. (1989b) and the Department of Chemical Engineering, University of Arkansas (1993).

At biological standard conditions, the formation of formic acid is scratch feasible. Increasing the partial pressure of hydrogen and carbon dioxide makes the reaction more favorable (Daniels, 1982), which in our opinion favors the direct hydrogenation of CO_2_ by the hydrogenase–formate-dehydrogenase complex. However, our results give that there is a non-linear relationship between formic acid formation and $p_{{\text{H}}_{2}}$. For we get 17 mM bar^−1^ when increasing $p_{{\text{H}}_{2}}$ to 2.13 bar (4 bar absolute pressure) and 25.6 mM bar^−1^ when increasing $p_{{\text{H}}_{2}}$ further to 3.73 bar which is contrary to what Peters et al. (1999) report. They state a linear relationship of 0.5 mM formic acid produced per 0.1 bar increase in $p_{{\text{H}}_{2}}$ for *A. woodii* and *A. carbinolicum* (Peters et al., 1999).

In our cultivations at 4 and 7 bar, $p_{{\text{H}}_{2}}$ is 2.13 and 3.73 bar but volumetric power input and gas feed rate is lower than the ones used by Kantzow and Weuster-Botz (2016). From the data in their publication a yield of formic acid per fed substrates of 0.002 g g^−1^ can be calculated which is 14 % of what is reported here at similar $p_{{\text{H}}_{2}}$ with a overall *Y*_P/S_ for formic acid of 0.015 g g^−1^. This indicates that, despite the differences between 1.5 L-scale and 2.5 L-scale, working with constant $\dot{n}V_{\text{L}}^{-1}$ yields a more substrate efficient process at elevated pressure than the classical approach of keeping ${\dot{V}}_{\text{g}}V_{L}^{-1}$ constant does. While the approach of constant volumetric gas feed rate ensures constant *k*_L_*a*-values if $PV_{L}^{-1}$ is kept constant as well (Schlüter et al., 1992) even at elevated pressure (Maier et al., 2001), ${\dot{V}}_{\text{g}}V_{L}^{-1}$ decreases with increasing pressure when $\dot{n}V_{\text{L}}^{-1}$ is kept constant. The actual volumetric flow rates for each pressure stage in this work are 0.029 vvm (1 bar), 0.007 vvm (4 bar), and 0.004 vvm (7 bar). Under these conditions, the *k*_L_*a*-value cannot assumed to be equal in all pressure stages. But since *k*_L_ is independent from pressure, an approximation for *k*_L_*a* at different pressures with $\dot{n}V_{\text{L}}^{-1}$ = constant can be calculated by

(3) $$k_{L}a_{\left(p_{2}\right)}={\left(\frac{p_{1}}{p_{2}}\right)}^{\frac{2}{3}}k_{L}a_{(p_{1}\text{)}}.$$

The deviation of this equation can be found in the Supplementary Material. This equation has also been used in the work of Linek and Sinkule (1991). Approximation of *k*_L_*a*-values for oxygen in medium with equation (3) results in 4.0 10^−3^ s^−1^ at 4 bar and 2.8 10^−3^ s^−1^ at 7 bar. *k*_L_*a*-values for different gases are proportional to each other by the square root of the quotient of their diffusion coefficients (Kodama et al., 1976). However, the formation of formic acid is more substrate efficient at higher pressures when $\dot{n}V_{\text{L}}^{-1}$ is kept constant although the gas–liquid mass transfer coefficient significantly decreases with increasing pressure.

## Conclusion

Our experiments with *C. ljungdahlii* show that complete consumption of fed substrates could not be achieved by increasing the absolute system pressure although no clear statement about actual substrate consumption at elevated pressures is possible due to the reasons discussed earlier. However, our data show that although the product spectrum changes at increased substrate pressure, the overall product yield from fed substrates is quite similar for all pressure stages examined in 2.5 L-scale.

Increasing the absolute system pressure and, therefore, the partial pressure of hydrogen and carbon dioxide results in a shift of the product spectrum and formic acid becomes a product of significance. On the other hand, biomass formation decreased with increasing substrate pressures. Whether this inhibition of biomass growth is subject to an inhibitory effect of increased hydrogen partial pressures as assumed by Kantzow and Weuster-Botz (2016) or more likely due to inhibitory effects of increased dissolved carbon dioxide (Eigenstetter and Takors, 2017) remains a topic of interest for further investigations. It might be possible that with a stepwise increase in process pressure with a steady built-up of biomass negative effects of increased substrate pressures are avoidable. This has already been shown by the Department of Chemical Engineering, University of Arkansas (1993) with *C. ljungdahlii* and carbon monoxide containing gases to avoid inhibitory effects of increased dissolved carbon monoxide concentrations.

The approach presented here uses constant $\dot{n}V_{\text{L}}^{-1}$ in all pressure stages and, therefore, the mass transfer coefficient decreases with each pressure step. However, yields of formic acid per fed substrate are 7.5 times higher than in processes with constant ${\dot{V}}_{\text{g}}V_{L}^{-1}$ as published by Kantzow and Weuster-Botz (2016).

Further investigations on the influence of feed gas flow rate on the fed substrate based yield are necessary to increase substrate efficiency of anaerobic fermentation of gaseous carbon and energy sources. The complete usage of substrates is a crucial point in increasing the overall efficiency of such processes.

## Nomenclature

**Table:** 

*β_I_*	Mass concentration of *i*
$\text{(}p_{\text{i}}^{\text{G}}-p_{\text{i}}^{\text{L}}\text{)}$	Driving force of mass transfer expressed as difference in partial pressure of *i* (bar)
(c* − *c*)	Difference of saturation concentration and actual concentration of a compound at process conditions (mol L^−1^)
*D*_1.5_	Inner diameter of reactor vessel in 1.5 L-scale (mm)
*d*_1.5_	Stirrer diameter in 1.5 L-scale (mm)
*D*_2.5_	Inner diameter of reactor vessel in 2.5 L-scale (mm)
*d*_2.5_	Stirrer diameter in 2.5 L-scale (mm)
*E*	Consumed amount of substance divided by the total fed amount of substance (%)
*H_i_*	Henry’s solubility coefficient of *i* (M bar^−1^)
*h*_l 2.5_	Filling level in 2.5 L-scale (mm)
*k*_L_*a*	Volumetric mass transfer coefficient (s^−1^)
$\dot{n}V_{\text{L}}^{-1}$	Volumetric amount of substance flow rate (mmol min^−1^ L^−1^)
$PV_{L}^{-1}$	Volumetric power input (W L^−1^)
$p_{{\text{CO}}_{2}}$	Partial pressure of carbon dioxide (bar)
$p_{H_{2}}$	Partial pressure of hydrogen (bar)
${\dot{V}}_{\text{g}}V_{L}^{-1}$	Volumetric gas flow rate (vvm)
*x_i_*	Amount of substance ratio calculated by *x*_i_ = *c_i_*/Σ*c_i_* (−)
*Y*_P/S_	Mass based product yield per substrate (g g^−1^)

## Author Contributions

FO: first idea and main design of experimental set-up for both scales. Scale-up from 1.5 to 2.5 L-scale. Supervision of experiments in 2.5 L-scale and execution of experiments in 1.5 L-scale. Sample analytics in all scales. Significant analysis and evaluation of results. Writing of the manuscript. IS: substantial performance of experiments and sample analytics in 2.5 L-scale. Critical revision of the manuscript. MZ: significant input on design of experimental set-up and supervision of experiments in 2.5 L-scale. Important input for evaluation of results and critical revision of the manuscript. SH: substantial input on reactor design and set-up of high-pressure vessel. Critical revision of the manuscript. JS: important support on the concept of the project and critical revision of the manuscript. NB: significant input on concept and experimental design in 2.5 L-scale. Critical revision of the manuscript. AN: substantial input on concept, experimental design, and evaluation of results. Critical revision of the manuscript.

## Conflict of Interest Statement

The authors declare that the research was conducted in the absence of any commercial or financial relationships that could be construed as a potential conflict of interest.
