# Supplementary material for: Formic Acid Formation by Clostridium ljungdahlii at Elevated Pressures of Carbon Dioxide and Hydrogen
Source: Front Bioeng Biotechnol. 2018 Feb 12;6:6. doi: 10.3389/fbioe.2018.00006 (PMC5816570; doi:10.3389/fbioe.2018.00006)
Supplement: Supplementary file 1 [file Data_Sheet_1.PDF]

# *Supplementary Material*

# Formic Acid Formation by *Clostridium ljungdahlii* at Elevated Pressures of Carbon Dioxide and Hydrogen

**Florian Oswald*, I. Katharina Stoll, Michaela Zwick, Sophia Herbig, Jörg Sauer, Nikolaos Boukis and Anke Neumann.**

*** Correspondence:** Florian Oswald: florian.oswald@kit.edu

## Derivation of Equation (3)

The mass transfer coefficient is defined as

$$k_{\mathrm{L}}a = k_{\mathrm{L}}\frac{A}{V_{\mathrm{L}}}.$$

With $A$ being the combined surface of all gas bubbles which may also be seen as one gas bubble with the surface $A$. The relationship between Surface of a gas bubble and pressure at given amount of substance is defined via the volume at each pressure. These are connected by the law of Boyle and Mariotte

$$V_2 = \frac{p_1}{p_2}V_1.$$

Appling the equation for the volume of a ball to the law of Boyle and Mariotte results in

$$d_2 = \sqrt[3]{\frac{p_1}{p_2}}d_1.$$

With $d_1$ and $d_2$ being the diameter of a gas bubble at pressures $p_1$ and $p_2$. The surface of a ball is given by $\pi d^2$ and therefore at different pressures

$$A_2 = \pi\left(\sqrt[3]{\frac{p_1}{p_2}}d_1\right)^2.$$

Which can be simplified into

$$A_2 = \left(\frac{p_1}{p_2}\right)^{\frac{2}{3}}A_1.$$

This represents the surface of a gas bubble with the same surface as all gas bubbles in the bioreactor together. Therefore the mass transfer coefficient at different pressures can be approximated by

$$k_{\mathrm{L}}a_{(p_2)} = \left(\frac{p_1}{p_2}\right)^{\frac{2}{3}} k_{\mathrm{L}}a_{(p_1)}.$$
